# Stimulated single-fiber electromyography in the diagnostic workup of diplopia and ptosis

**DOI:** 10.1016/j.cnp.2026.09.004

**Published:** 2026-09-19

**Authors:** Sara Cornacchini, Massimiliano Ugo Verza, Gregorio Spagni, Francesca Beretta, Antonio Farina, Maria Bastianelli, Annalisa Cassardo, Cristina Mei, Antonio Lotti, Alessandro Barilaro, Luca Massacesi, Valentina Damato, Antonello Grippo

**Affiliations:** a Department of Neurosciences Drugs and Child Health, University of Florence, Florence, Italy; b Department of Neurology 2, Careggi University Hospital, Florence, Italy; c Reference Centre for Paraneoplastic Neurological Syndromes and Autoimmune Encephalitis, Hospices Civils de Lyon, Neurological Hospital, Bron, France; d MeLis, UCBL-CNRS UMR 5284, INSERM U1314, Université Claude Bernard Lyon 1, Lyon, France; e Department of Neurophysiology, Careggi University Hospital, Florence, Italy

**Keywords:** Single fiber electromyography, Myasthenia gravis, Diplopia, Ptosis, SFEMG, MG

## Abstract

**Objective:**

Ptosis and diplopia are common neuro-ophthalmological presentations with a broad differential diagnosis. In clinical practice, stimulated single-fiber electromyography (SFEMG) is often requested when ocular myasthenia gravis (MG) remains a diagnostic consideration after initial evaluation. This study evaluated the diagnostic contribution of stimulated SFEMG in a real-world cohort of patients referred with ptosis and/or diplopia.

**Methods:**

Patients presenting with ptosis, diplopia or both were prospectively recruited and underwent comprehensive serological, radiological, and electrophysiological assessment, including SFEMG. Normative cut-offs for stimulated concentric needle SFEMG were established from 31 healthy controls and applied to the study population.

**Results:**

A total of 115 patients were included (diplopia *n* = 46, ptosis *n* = 25, both *n* = 44). MG was diagnosed in 46 patients. SFEMG showed a sensitivity of 60.9% (95% CI 0.45–0.75) and specificity of 92.8% (95% CI 0.84–0.98), with a positive predictive value of 84.9% and negative predictive value of 78.1%. SFEMG identified 7 seronegative MG cases and 16 cases with negative RNS. In the non-MG group (69/115), SFEMG was negative in 92.8%, supporting its role in excluding MG.

**Conclusions:**

Stimulated SFEMG demonstrates high specificity in patients with ptosis and diplopia and is particularly useful in diagnostically challenging cases, including seronegative MG and RNS-negative patients.

**Significance:**

SFEMG represents a valuable second-line diagnostic tool in suspected ocular MG. Its high specificity supports its use in selected patients to confirm diagnosis and reduce the risk of MG overdiagnosis and unnecessary treatment.

## Highlights

• SFEMG showed high specificity but moderate sensitivity for ocular MG diagnosis.
• SFEMG supported MG diagnosis in seronegative and RNS-negative selected patients.
• Local SFEMG cut-offs and clinical context are essential to avoid MG overdiagnosis.

## 1 Introduction

Single fiber electromyography (SFEMG) is a specialized technique that demands considerable expertise (Sanders et al., 2019). It is mainly used in tertiary referral centers as a second-level diagnostic tool for the evaluation of patients with suspected myasthenia gravis (MG) when first-line tests are inconclusive. This is especially relevant in patients with ocular symptoms, such as ptosis and/or diplopia. These neuro-ophthalmological manifestations are commonly encountered in clinical practice and may result from various underlying conditions, making the diagnosis particularly challenging (Díaz-Manera et al., 2018; Gräf and Lorenz, 2012). SFEMG has long been considered the most sensitive electrophysiological test for the diagnosis of MG (Evoli et al., 2019); however, recent studies have shown that its performance in patients with solely ocular symptoms is characterized by variable estimates of the sensitivity and specificity of SFEMG (Benatar et al., 2006; Giannoccaro et al., 2020; Morren et al., 2016). The objective of this study was to prospectively evaluate the accuracy of the stimulated SFEMG in supporting the diagnosis of MG in patients presenting with diplopia and/or ptosis.

## 2 Methods

### 2.1 Study population

The study focused on the diagnostic contribution of stimulated SFEMG in patients referred for ptosis and/or diplopia with suspected MG. We conducted a real-world, single-center observational study including consecutive patients prospectively evaluated in routine clinical practice for isolated or combined ptosis and diplopia as their initial neurological manifestation and referred to the MG Clinic at AOU Careggi between 2016 and 2025. Patients underwent standardized clinical, serological, radiological, and electrophysiological assessment, including stimulated SFEMG. Orthoptic evaluation, thyroid function testing, and ice pack test were also assessed when appropriate (Fig. 1). Antibody testing was performed according to the best available clinical practice at the time of evaluation. Anti-AChR and anti-MuSK antibodies were assessed by RIA throughout the study period; live cell-based assay (CBA) for clustered AChR, MuSK, and LRP4 antibodies was introduced subsequently and was performed in seronegative cases when available and clinically indicated (Damato et al., 2022). Repetitive nerve stimulation (RNS) was conducted on distal (abductor digiti minimi), proximal (trapezius), and facial muscles. When clinically indicated, a standardized pyridostigmine trial (60 mg four times daily for at least one week) was administered. Clinical response was assessed using MG-ADL or QMG scores. Pyridostigmine was discontinued ≥12 h before electrophysiological testing. Genetic testing for congenital myasthenic syndrome (CMS) was performed in patients with clinical suspicion of CMS using a targeted next-generation sequencing panel including *DOK7, MUSK, GFPT1, DPAGT1,* and *CHRND*. The patient had autosomal recessive CHRND-related CMS, with two heterozygous CHRND variants identified by genetic testing. CMS was considered in seronegative patients with clinical features suggestive of a congenital neuromuscular junction disorder, including early onset, long-standing symptoms, family history, atypical phenotype, or poor/absent response to immunotherapy.

**Fig. 1:**
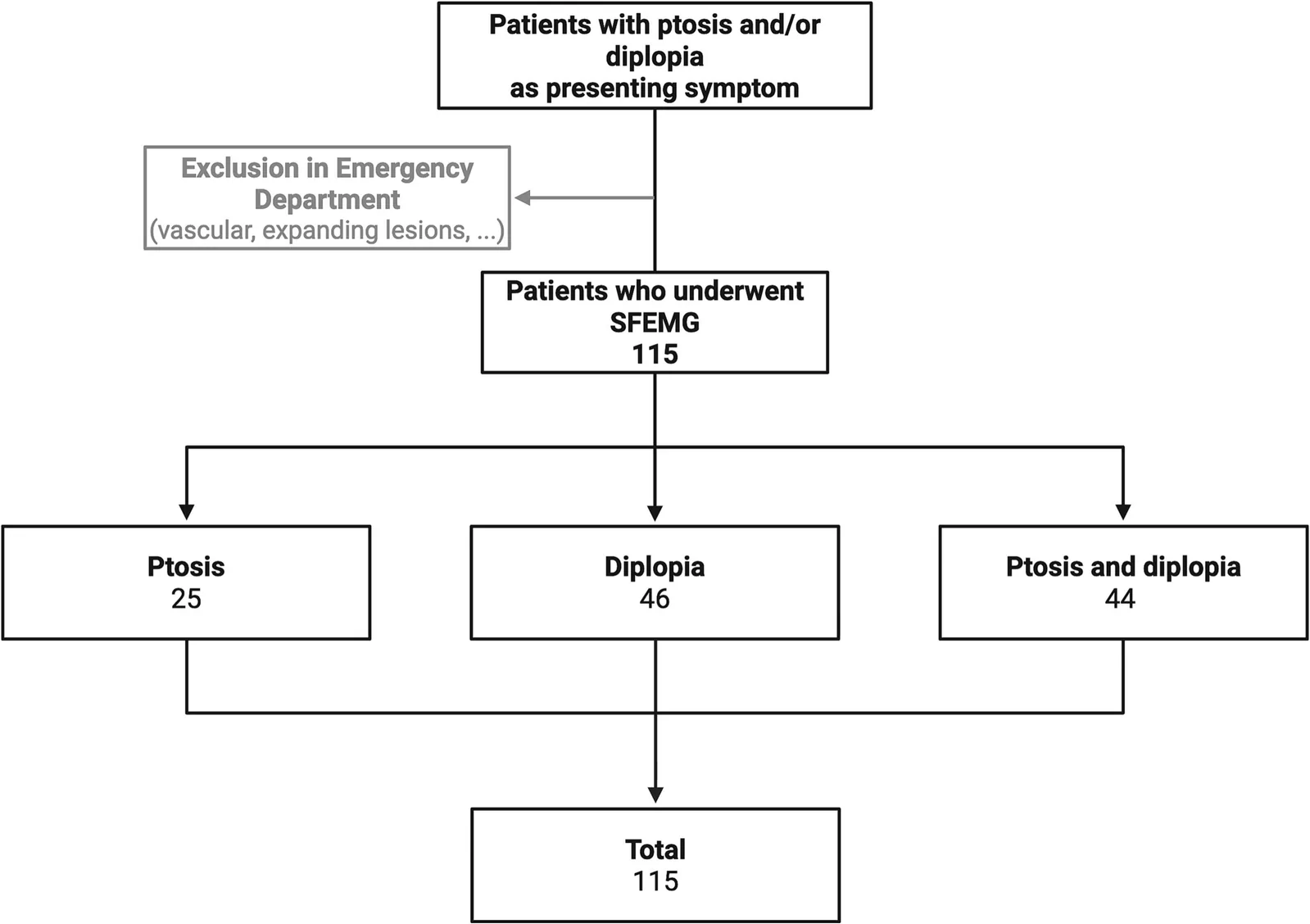
Flowchart of patient recruitment. Patients presenting with ptosis and/or diplopia underwent initial emergency department evaluation to exclude vascular or space-occupying lesions. A total of 115 patients were subsequently assessed with SFEMG, including 25 with isolated ptosis, 46 with isolated diplopia, and 44 with both ptosis and diplopia. Abbreviations: SFEMG: single-fiber electromyography.

Final diagnosis was reviewed at study closure and adjudicated using all available clinical, serological, electrophysiological, radiological, and follow-up data by MG experts (FB, AB, VD) blinded to SFEMG results. SFEMG positivity was not used as a diagnostic criterion. Final diagnoses other than MG, including blepharospasm, eyelid laxity, vascular disorders, thyroid eye disease, neurodegenerative disorders, and functional disorders, were assigned only after completion of diagnostic workup and follow-up.

### 2.2 Ethical board approval

The present research analysis was approved by the local Ethics Committee of AOU Careggi, Florence, Italy, under protocol n. 22914_bio, approved on 10/02/2023. The protocol covered the use of prospectively collected clinical data from patients evaluated in routine clinical practice for ptosis and/or diplopia and the continuation of patient inclusion after approval. Written informed consent and/or authorization for the use of anonymized clinical data was obtained according to the requirements of the approved protocol and applicable institutional regulations.

### 2.3 SFEMG recording techniques

Stimulated SFEMG was performed using a concentric needle electrode (0.30 mm diameter; recording area 0.019 mm^2^; Ambu, Denmark) and Synergy EMG system (Natus Medical). The frontalis muscle was selected due to its superficial location, procedural tolerability, and reproducibility (Rouseev et al., 1992; Valls-Canals et al., 2003; Valls-Canals et al., 2000). Stimulation of the temporal branch of the facial nerve was delivered at 10 Hz (3–8 mA) to elicit minimal visible contraction. At least 20 acceptable endplates from ≥3 insertions were analyzed per patient, following established guidelines (Sanders et al., 2022; Sanders et al., 2019; Trontelj and Stålberg, 1992). All examinations were performed by a single experienced neurophysiologist blinded to final diagnosis.

SFEMG was requested when ptosis, diplopia, or eyelid-opening symptoms raised clinical suspicion of a neuromuscular junction disorder. Clinical features supporting referral included fluctuating ptosis and/or diplopia, fatigability, diurnal variability, fatigable eyelid weakness, orbicularis oculi weakness or Cogan lid twitch when present, unexplained ocular misalignment, negative or inconclusive first-line investigations despite persistent clinical suspicion, or referral for suspected ocular MG after exclusion of urgent structural or vascular causes.

### 2.4 Parameters analyzed

The following parameters were analyzed:

• Mean consecutive difference (MCD)
• Percentage of endplates with abnormal jitter
• Impulse blocking

SFEMG was considered abnormal when either:

• Mean MCD > 26 μs
• Individual jitter >32 μs in ≥27% of endplates
• Impulse blocking alone was not considered diagnostic.

### 2.5 Calculation of normative values

Local normative values were derived from 31 healthy volunteers (54.8% female; mean age 43 ± 18 years). Controls were not age-matched to patients. All participants were screened for prior botulinum toxin exposure and neuromuscular disease. Mean MCD was normally distributed; therefore, the upper local reference limit was defined as mean + 2 SD, consistent with previous studies (Damato et al., 2022; Stålberg et al., 2016), yielding 26.13 μs, rounded to 26 μs (Supplementary Fig. 1A). Individual jitter values were not normally distributed; therefore, the 95th percentile of values obtained from healthy controls was used, yielding 32.18 μs, rounded to 32 μs (Supplementary Fig. 1B). The proportion of endplates with jitter >32 μs was then evaluated in the symptomatic cohort as a diagnostic classifier. Because the conventional 10% threshold for abnormal endplates produced substantial overlap between MG and non-MG patients in our cohort, we explored a cohort-derived diagnostic threshold aimed at improving specificity. The 27% threshold was therefore considered an exploratory, specificity-oriented diagnostic decision threshold derived from the present clinical cohort, not a normative reference limit (Supplementary Fig. 1C).

Fare clic o toccare qui per immettere il testo.Impulse blocking was absent in healthy controls. In symptomatic patients, it was frequent in MG when associated with other abnormalities but also occurred in non-MG cases when isolated; therefore, isolated impulse blocking was not considered diagnostic. Stimulated frontalis SFEMG was considered abnormal if either:

a. mean MCD > 26 μs, or.
b. individual jitter >32 μs in ≥27% of endplates.

### 2.6 Statistical analysis

Statistical analyses were performed using SPSS v29. Continuous variables were summarized as mean ± SD or median (IQR) as appropriate. Group comparisons used Student's *t*-test or Mann–Whitney *U* test. Categorical variables were compared using Chi-square or Fisher's exact test. McNemar's test was used for paired categorical comparisons. Diagnostic performance was expressed as sensitivity, specificity, positive predictive value (PPV), negative predictive value (NPV), likelihood ratios (LR+ and LR−), and pos*t*-test probability (95% CI). ROC curves were used to evaluate the discriminatory performance of SFEMG parameters. Two-sided *p* < 0.05 was considered statistically significant. The distribution of normative values was assessed for normality using the Shapiro–Wilk test.

## 3 Results

### 3.1 Application of SFEMG in the diplopia and ptosis cohort

A total of 115 patients presenting with ptosis and/or diplopia as the initial neurological symptom were prospectively included in the study. Among them, 25 presented with isolated ptosis, 46 with isolated diplopia, and 44 with both ptosis and diplopia (Fig. 1). In this cohort 61 were females (53.0%), the mean age at symptom onset was 58,3 years (± 18,1 (SD); range 17–87). All patients underwent SFEMG. The mean age at the time of SFEMG was 60.6 years (± 16.7 (SD); range 17–87), with an average delay of 2.2 years between symptom onset and SFEMG evaluation. The final diagnoses were very heterogeneous and are summarized in Fig. 2. Interestingly, patients who presented with both ptosis and diplopia had a higher likelihood of being diagnosed with MG [25/44 (56.8%)]. In contrast, those presenting with isolated diplopia were more frequently diagnosed with non-MG conditions, while only 10/46 (21.7%) patients were diagnosed with MG. Patients presenting with isolated ptosis represented an intermediate scenario, with 11/25 cases (44%) ultimately diagnosed with MG. McNemar's test showed that the likelihood of MG diagnosis was significantly higher in patients with ptosis and diplopia compared with those presenting with isolated diplopia (χ^2^ = 5.22, *p* = 0.021), and in patients with ptosis compared with those with isolated diplopia (χ^2^ = 9.68, *p* = 0.001), whereas no significant difference was found between the “ptosis” and the “ptosis+diplopia” groups. Overall, SFEMG was positive in 33 out of 115 patients (28.7%). When SFEMG results were stratified by presenting symptom, the highest positivity rate was observed in patients with both ptosis and diplopia (38.6%) (Chi-square test *p* = 0.077 – Fisher's exact test *p* = 0.073, Table 1).

**Fig. 2:**
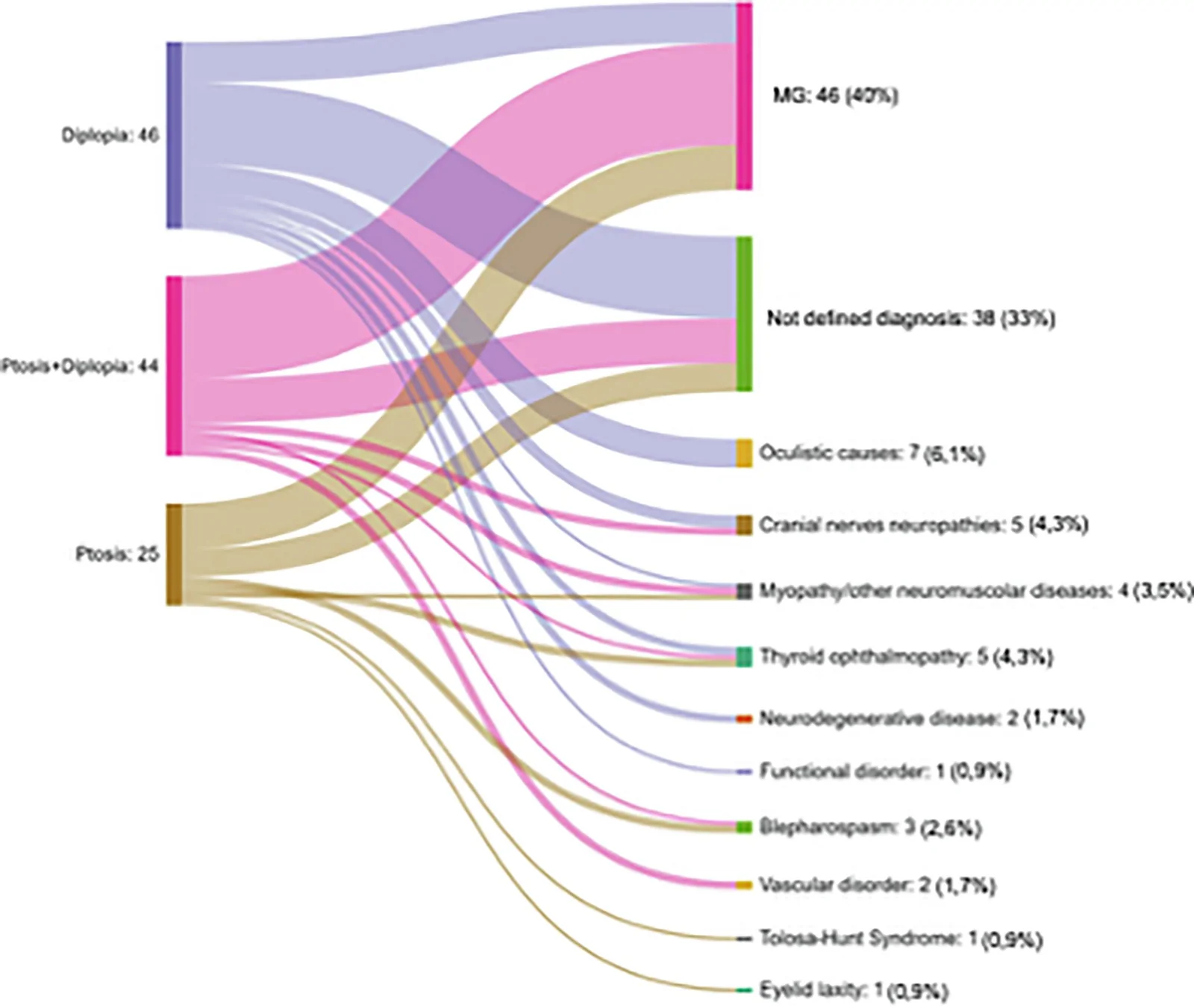
Final diagnoses of patients recruited in the study. Sankey diagram illustrating the distribution of presenting symptoms — ptosis, diplopia, or both — SFEMG findings, and corresponding final diagnoses. MG was the most frequent diagnosis (46/115, 40%), followed by cases without a defined diagnosis (NDD, not defined diagnosis; 38/115, 33%). Other etiologies included ophthalmologic causes, cranial nerve neuropathies, myopathy/other neuromuscular diseases, thyroid ophthalmopathy, neurodegenerative diseases, functional disorders, blepharospasm, vascular disorders, Tolosa–Hunt syndrome, and eyelid laxity, with proportions indicated in the figure. Among patients without a final diagnosis of MG, abnormal SFEMG findings were observed in patients with NDD and in selected non-MG diagnostic categories, as shown by the flow from SFEMG-positive results to final diagnoses. Abbreviations: MG: myasthenia gravis, NDD: not defined diagnosis.

**Table 1:** SFEMG results stratified by presenting symptoms.

	Ptosis	Diplopia	Ptosis and diplopia
SFEMG +	8 (32%)	8 (17.4%)	17 (38.6%)
SFEMG -	17 (68%)	38 (82.6%)	27 (61.4%)
Total	25	46	44

Chi-square test *p* = 0.077 – Fisher's exact test *p* = 0.073.

Abbreviations: SFEMG: single-fiber electromyography.

### 3.2 SFEMG performance in MG detection

When evaluating the diagnostic performance of SFEMG in relation to the final diagnosis of MG, the test was positive in 28 out of 46 MG patients (60.9%) (Table 2, Fig. 3). Among non-MG patients, SFEMG was negative in the majority of cases (64 out of 69, 92.8%), with only 5 SFEMG-positive non-MG patients (7.2%) (Chi-Square test *p* < 0.001), who had the following diagnoses: one blepharospasm whose symptoms improved with clonazepam in the follow up, one thyroid eye disease, and three cases with undetermined final diagnoses. Among the three patients with abnormal SFEMG and undetermined final diagnosis, additional investigations included brain/orbital MRI, orthoptic evaluation, thyroid function testing, antibody testing, RNS and a long follow up. Muscle biopsy was not performed in these patients; therefore, mitochondrial myopathy or other rare myopathic conditions cannot be definitively excluded. CMS was considered when clinically appropriate. Two of the three patients underwent targeted NGS testing for CMS-related genes, including DOK7, MUSK, GFPT1, DPAGT1, and CHRND, with negative results. One additional patient with undetermined final diagnosis was not genetically tested; therefore, a rare congenital neuromuscular junction disorder couldn't be completely excluded.

**Table 2:** Diagnostic concordance of SFEMG with other diagnostic tests.

	MG	Non-MG	Post test probability	*p* value
**SFEMG +**	28 (60.9%)	5 (7.2%)	84.8%	<0.001
**SFEMG -**	18 (39.1%)	64 (92.8%)		
Tot patients tested	46	69		
**AB+**	36 (78.3%)	2 (2.9%)	94.7%	<0.001
**AB -**	10 (21.7%)	67 (97.1%)		
Tot patients tested	46	69		
**RNS+**	12 (27.9%)	0 (0%)	≈ 100%	<0.001
**RNS -**	31 (72.1%)	65 (100%)		
Tot patients tested	43	65		
**PYR+**	34 (89.5%)	6 (35.3%)	63%	<0.001
**PYR -**	4 (10.5%)	11 (64.7%)		
Tot patients tested	38	17		
**Ice pack test +**	9 (100%)	4 (66.7%)	69.2%	0.42
**Ice pack test -**	0 (0%)	2 (33.3%)		
Tot patients tested	9	6		

Abbreviations: MG: myasthenia gravis; SFEMG: single-fiber electromyography; AB: MG-antibodies test; RNS: repetitive nerve stimulation; PYR: pyridostigmine trial.

**Fig. 3:**
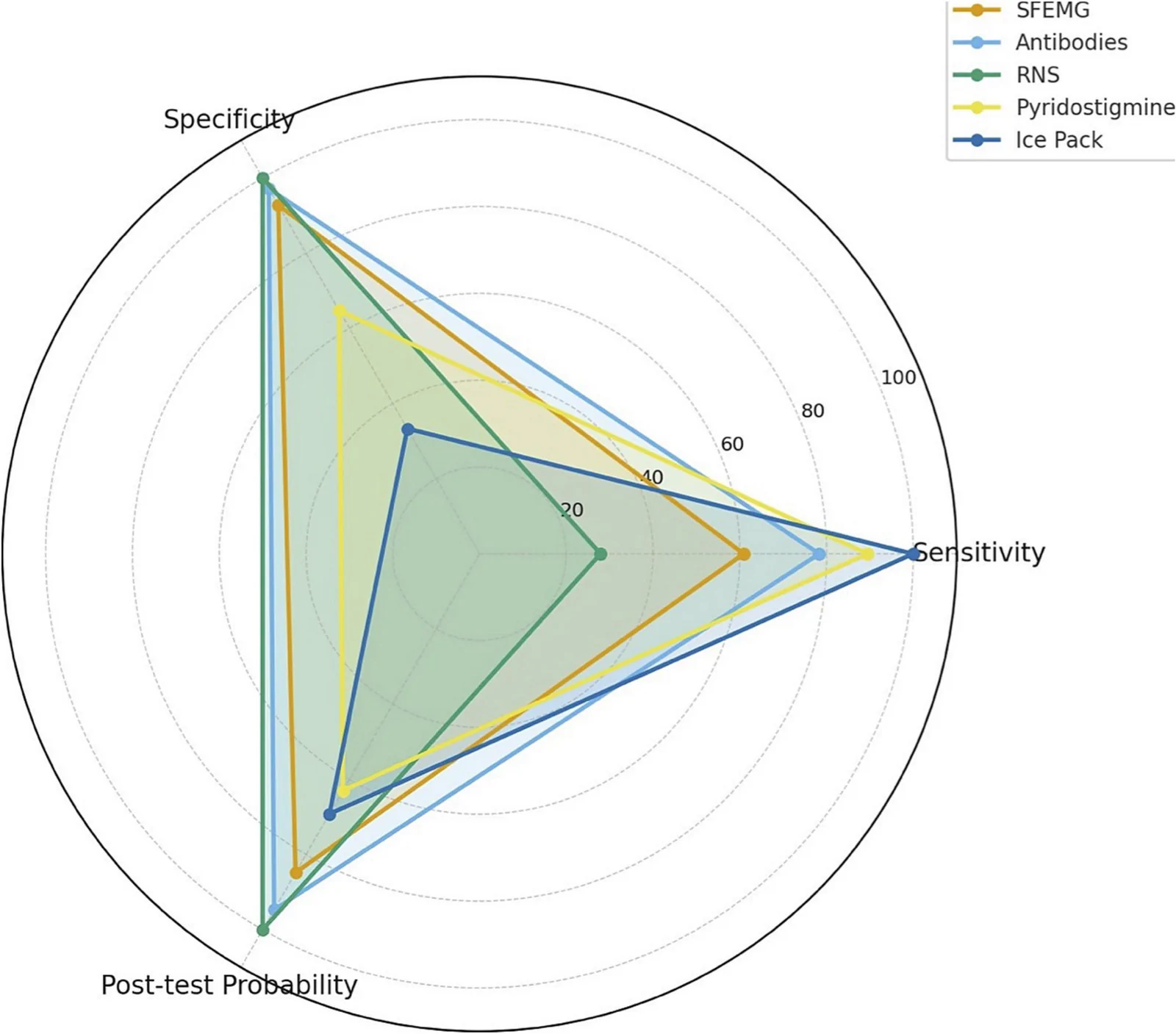
Radar chart of diagnostic performance profiles of MG tests. Each axis represents a diagnostic parameter (sensitivity, specificity, and post-test probability). The performance of SFEMG, antibody testing, RNS, pyridostigmine test, and ice pack test are compared. The plot highlights the relative strengths and limitations of each method across the different diagnostic dimensions. Abbreviations: MG: myasthenia gravis; SFEMG: single-fiber electromyography; RNS: repetitive nerve stimulation.

Overall, SFEMG was positive in 7/10 (70%) patients with seronegative MG and in 16/43 (37.2%) MG patients with negative RNS. Among these, five SFEMG-positive patients had negative antibody testing and negative RNS. Their final diagnosis of MG was based on the overall clinical picture, including fluctuating ocular symptoms, exclusion of alternative diagnoses during follow-up, and clinically meaningful response to pyridostigmine. Pyridostigmine response alone was not considered sufficient to establish the diagnosis. CMS was considered when clinically appropriate. Four patients underwent targeted NGS testing for CMS-related genes, including DOK7, MUSK, GFPT1, DPAGT1, and CHRND, with negative results. The remaining patient was not genetically tested; therefore, a rare congenital neuromuscular junction disorder couldn't be completely excluded. In clinical practice, SFEMG represented the only abnormal instrumental test supporting impaired neuromuscular transmission in these cases.

### 3.3 Comparison of SFEMG parameter alterations between MG and non-MG cohorts

Among patients with MG who exhibited a positive SFEMG, the number of altered parameters — defined as a mean MCD > 26 μs, an individual jitter >32 μs in at least 27% of endplates, and the presence of impulse blocking — was as follows: one parameter was altered in 2 of 28 patients (7.1%), two parameters in 9 of 28 patients (32.1%), and all three parameters in 17 of 28 patients (60.7%). In the subgroup of non-MG patients with a positive SFEMG, 3 of 5 patients showed only one altered parameter, whereas 1 patient had two, and 1 patient had all three parameters altered (MG vs non-MG: Chi-Square test p 〈0,001) (Fig. 4A). Mean MCD was significantly higher in MG patients [30.08 ± 11.92 (SD)] compared with non-MG patients [19.01 ± 4.30 (SD)] (independent-samples Student's *t*-test, t(53.9) = −6.40, *p* < 0.001). The mean difference was 11.06 (95% CI 7.59 to 14.53) (Fig. 4B). Mean MCD was significantly higher in MG patients with a positive SFEMG [35.69 ± 10.87 (SD)] compared with MG patients with a negative SFEMG [21.34 ± 3.31 (SD)] (independent-samples Student's *t*-test, t(34.2) = −6.53, *p* < 0.001). In non-MG patients with a negative SFEMG mean MCD was 18.39 ± 3.73 (SD), whereas in non-MG patients with a positive SFEMG it was 26.97 ± 3.12 (SD) (independent-samples Student's t-test, t(4.9) = −5.83, *p* = 0.002). The percentage of endplates with abnormal jitter (>32 μs) was 32.05% ± 23.66 in MG patients and 9.51% ± 8.94 in non-MG patients. The difference was statistically significant (Mann–Whitney *U* test, U = 2654, *p* < 0.001).

**Fig. 4:**
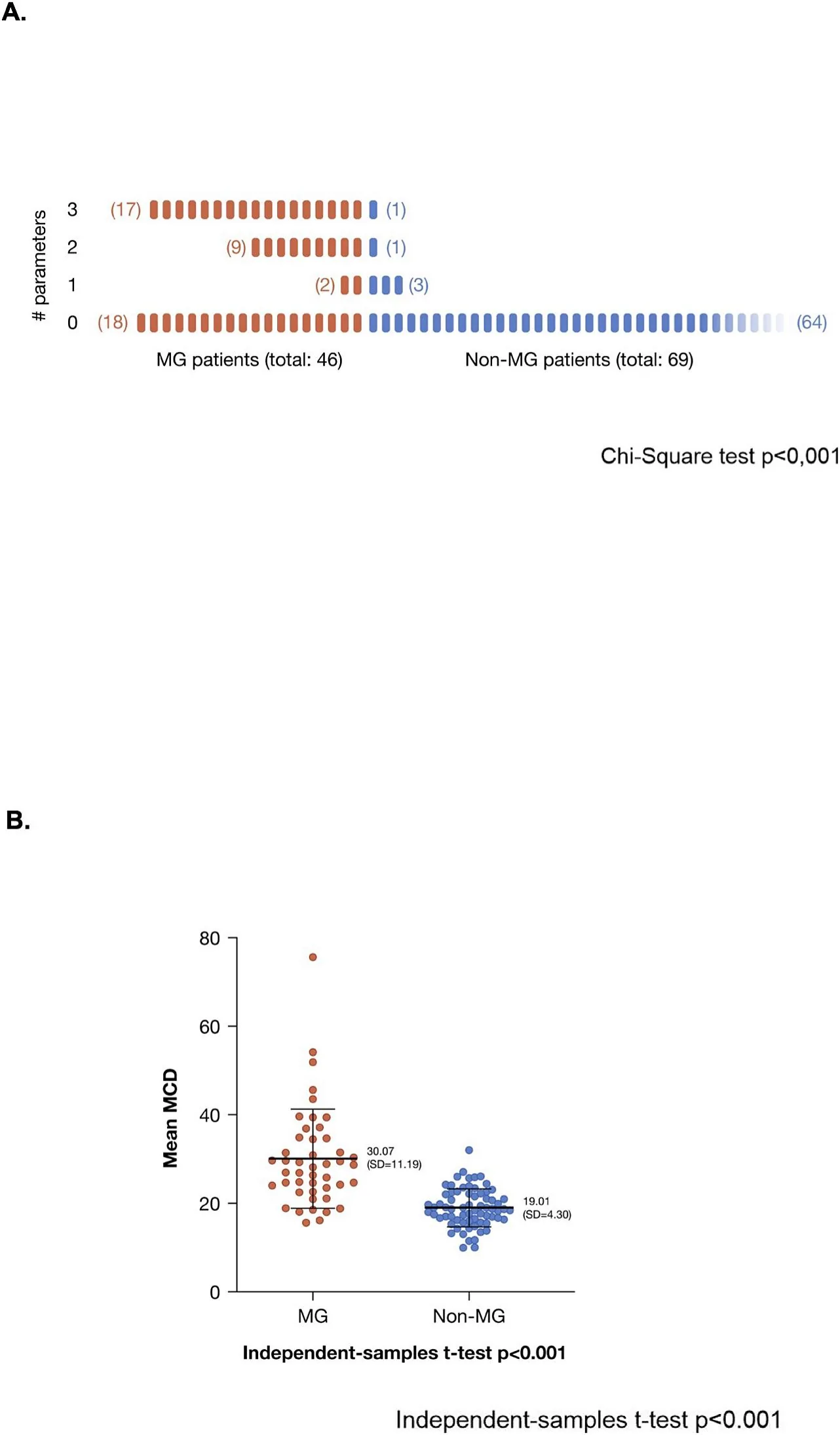
Comparison of SFEMG parameter alterations between MG and non-MG cohorts. (A) Distribution of the number of altered parameters, defined as mean MCD > 26 μs, individual jitter >32 μs in ≥27% of endplates, or impulse blocking. Most MG patients with a positive SFEMG showed two or three altered parameters, whereas non-MG patients more frequently had only one (Chi-Square test *p* < 0.001). (B) Mean MCD values were significantly higher in MG patients [30.08 μs ± 11.92 (SD)] than in non-MG patients [19.01 μs ± 4.30 (SD)] (Independent-samples Student's t-test, t(53.9) = −6.40, *p* < 0.001). Abbreviations: MG: myasthenia gravis; MCD: mean consecutive difference; SFEMG: single-fiber electromyography.

### 3.4 Descriptive concordance between SFEMG, ancillary diagnostic tests, and final clinical diagnosis

MG antibody testing with RIA and, if negative, with live CBA, was positive in 36 out of 46 patients with MG (78.3%), while 10 patients (21.7%) were seronegative, including one patient with genetically confirmed *CHRND*-related congenital myasthenic syndrome (Table 2). (Table 2). Among the seropositive patients, 33 were positive for anti-AChR antibodies, 2 for anti-MuSK, and 1 for anti-clustered AChR. In the non-MG cohort, only two false positives were observed (2.9%) (Chi-square test, *p* < 0.001). Both cases were analyzed by RIA, yielding one anti-AChR–positive result (4 nmol/L) and one anti-MuSK–positive result (0.37 nmol/L). The latter was further evaluated with live CBA, which resulted negative. RNS test was performed in 108/115 (93.9%) subjects. Among MG patients, RNS resulted negative in 31/43 (72.1%) and positive in 12/43 (27.9%). Importantly, all MG patients with positive RNS test developed generalized MG between onset and RNS testing or shortly after RNS testing, with the only exception of the patient with genetically confirmed autosomal recessive CHRND-related CMS, who had persistently ocular symptoms. In the non-MG cohort, all subjects had a negative RNS test (65/65, 100%) (Chi-Square test *p* < 0.001) (Table 2). A trial with pyridostigmine was performed in 55/115 (47.8%) subjects. In the MG cohort, 34/38 (89.5%) responded to pyridostigmine administration, while 4/38 (10.5%) did not respond. In the non-MG cohort, 6/17 (35.3%) reported an improvement of ptosis or diplopia after pyridostigmine administration, while 11/17 (64.7%) did not respond (Chi-Square test *p* < 0.001) (Table 2). Finally, the ice pack test was performed in 15/115 (13.0%) patients with ptosis, leading to an improvement of the ptosis in all MG patients (9/9, 100%), but also in 4/6 (66.7%) non-MG patients, while 2/6 had a negative test (33.3%) (Chi-Square test-*p* = 0.42 – Fisher's exact test *p* = 0.143) (Table 2). Assuming the same pre-test probability of 40%, as observed in this cohort, the pos*t*-test probability of MG varied according to the diagnostic test used. Following a positive result, the post-test probability was 84.8% for SFEMG, 94.7% for antibody testing, nearly 100% for RNS, 63.0% for response to pyridostigmine, and 69.2% for the ice pack test (Table 2, Fig. 3). Because antibody status, RNS, pyridostigmine response, and ice-pack test could contribute to final clinical adjudication, their diagnostic performance should be interpreted descriptively and may be affected by incorporation bias. In contrast, SFEMG was not used as a diagnostic criterion, and adjudicators were blinded to SFEMG results.

### 3.5 Diagnostic accuracy of SFEMG in MG

SFEMG showed a sensitivity of 60.9% (95% CI: 0.45–0.75) and a specificity of 92.8% (95% CI: 0.84–0.98). The PPV was 84.9% (95% CI: 0.70–0.93), while the NPV was 78.1% (95% CI: 0.71–0.84). Moreover, the diagnostic accuracy of SFEMG in identifying MG was assessed using ROC curve analysis. For the mean MCD, the area under the curve (AUC) was 0.863 (95% CI: 0.793–0.934). For the percentage of abnormal endplates, the AUC was 0.836 (95% CI: 0.762–0.911) (Supplementary Fig. 2). When applying the threshold values suggested in the literature by Sanders et al. (Stålberg et al., 2016) for the interpretation of SFEMG, the test showed a sensitivity of 95.65% (95% CI: 85.16%–99.47%) and a specificity of 53.62% (95% CI: 41.20%–65.72%) (Supplementary Table 1).

## 4 Discussion

In this real-world cohort of patients presenting with ptosis, diplopia, or both, stimulated SFEMG of the frontalis muscle showed high specificity but moderate sensitivity for the final diagnosis of MG. These findings support its role as a confirmatory neurophysiological test in clinically selected patients in whom ocular MG remains part of the differential diagnosis. Importantly, SFEMG was not used as a criterion for final diagnostic adjudication, which was based on clinical features, antibody testing, RNS, treatment response when available, exclusion of alternative diagnoses, and follow-up. The diagnostic contribution of SFEMG was particularly relevant in patients with negative or inconclusive first-line investigations, including antibody-negative cases.

Our findings are consistent with previous studies showing that SFEMG is highly sensitive for disorders of the neuromuscular transmission, but that its diagnostic specificity depends strongly on patient selection, muscle examined, technical methodology, and the criteria used to define abnormality (Benatar et al., 2006; Cosentino et al., 2024; Giannoccaro et al., 2020; Lo et al., 2017; Mercelis and Merckaert, 2011; Morren et al., 2016; Padua et al., 2014; Padua et al., 2000; Rouseev et al., 1992; Valls-Canals et al., 2003; Valls-Canals et al., 2000). A comparative summary of the main published SFEMG studies is provided in Table 3.

**Table 3:** Comparative summary of the main SFEMG studies reported in literature. Values from the present study are shown in the last row for direct comparison.

	SFEMG Sensitivity	SFEMG Specificity	Number of patients/ Type of recruitment	Type of activation	Type of needle	Muscles studied
Padua (2000) ^15^	100%	89%	86 Prospective	Voluntary activation	SFEMG needle electrode	Orbicularis oculi 84/86 cases, Orbicular oris 2/86 cases
Benatar (2006) ^7^	62%	96%	51 Retrospective	Voluntary activation	Concentric needle	Frontalis
Mercelis (2010) ^17^	80%	97%	456 Retrospective	Stimulated activation	SFEMG needle electrode	Orbicularis oculi
Padua (2014) ^16^	98%	70%	100 Prospective	Voluntary activation	SFEMG needle electrode	Orbicularis oculi
Morren (2016) ^6^	78% (For OMG 73%)	91% (For OMG 91%)	348 Retrospective	Voluntary activation	SFEMG needle electrode	Orbicularis oculus (89%), Frontalis (10%), Extensor digitorum communis (4%)
Lo et al. (2017)^18^	94% 97%	17% 22%	99 MG 61 MG Prospective	Stimulated activation	SFEMG needle electrode	Orbicularis oculi
Giannoccaro (2019) ^5^	79%	80%	232 Prospective	Stimulated activation	SFEMG needle electrode	Orbicularis oculi
Cosentino (2024) ^19^	79%	89%	34 Prospective	Stimulated activation	Concentric needle	Orbicularis oculi
Our study (2026)	60.9%	92.8%	115 Prospective	Stimulated activation	Concentric needle	Frontalis

Abbreviations: SFEMG: single-fiber electromyography.

In ocular presentations, diagnostic interpretation is especially challenging because ptosis and diplopia may result from several neurological, ophthalmological, endocrine, vascular, or functional disorders (Díaz-Manera et al., 2018; Gräf and Lorenz, 2012). Previous real-world neurophysiological cohorts of patients referred for isolated ocular symptoms similarly reported that only a proportion of patients ultimately receive a diagnosis of MG, while many receive alternative diagnoses or remain undiagnosed (Benatar et al., 2006; Giannoccaro et al., 2020; Lo et al., 2017; Mercelis and Merckaert, 2011; Morren et al., 2016; Padua et al., 2014; Valls-Canals et al., 2003). In this context, the inclusion of non-MG final diagnoses in our cohort should not be interpreted as inappropriate patient selection, but rather as reflecting the clinical setting in which SFEMG is actually used: to distinguish ocular MG from relevant mimics. Blepharospasm and related eyelid-opening disorders are examples of such diagnostic pitfalls, as MG may present with blinking or eyelid-opening symptoms mimicking blepharospasm, and conversely in patients with primary blepharospasm, MG has been reported as one of the conditions most frequently associated with diagnostic confusion (Fionda et al., 2026; Huang et al., 2015; Naum and Morena, 2025; Roberts et al., 2002; Zambelis et al., 2015).

A key message of our study is that SFEMG should not be used as an indiscriminate screening test in all patients with ptosis or diplopia. Rather, its diagnostic value is greatest when applied after careful clinical assessment in patients with a plausible suspicion of ocular MG, particularly when antibody testing and RNS are negative or inconclusive. The high specificity observed in our cohort may help reduce overdiagnosis of MG in patients with ocular symptoms, an important issue because abnormal jitter is not entirely disease-specific and may occur in other neuromuscular or local muscle conditions. For this reason, we avoided interpreting isolated SFEMG abnormalities as sufficient for MG diagnosis (Andrapalliyal et al., 2023). In patients without detectable antibodies, current recommendations emphasize the need for neurophysiological support and exclusion of alternative diagnoses. Our data support this approach and highlight the role of stimulated SFEMG as part of a composite diagnostic process rather than as a standalone diagnostic criterion.

This study has limitations. First, it was conducted in a single tertiary referral centre with substantial SFEMG expertise. This ensured technical consistency but may limit generalizability to less experienced settings. Second, the healthy-control group was modest in size; therefore, the present study should not be interpreted as establishing universal normative values for stimulated frontalis SFEMG. The control group was used to define local reference values for mean MCD and individual jitter, whereas the 27% abnormal-endplate threshold was cohort-derived and requires external validation. Third, antibody testing evolved during the long inclusion period, particularly with the later availability of live CBA, which may have introduced some heterogeneity in seronegative classification. Finally, in patients with abnormal SFEMG but without final MG diagnosis, rare alternative causes of increased jitter, including mitochondrial or other myopathic disorders, cannot be completely excluded when muscle biopsy or genetic testing was not performed.

In conclusion, stimulated SFEMG of the frontalis muscle is a valuable second-line diagnostic tool in patients presenting with ptosis and/or diplopia when ocular MG remains clinically suspected after initial evaluation. In this real-world tertiary referral cohort, SFEMG showed high specificity but moderate sensitivity, supporting its role in confirming impaired neuromuscular transmission in selected patients rather than as a general screening test. Interpretation should consider local methodology, clinical context, and the possibility of abnormal jitter in non-MG conditions.

## CRediT authorship contribution statement

**Sara Cornacchini:** Conceptualization, Investigation, Data curation, Formal analysis, Methodology, Writing – original draft. **Massimiliano Ugo Verza:** Data curation, Writing – original draft. **Gregorio Spagni:** Resources, Methodology, Supervision, Validation, Writing – review & editing. **Francesca Beretta:** Resources, Methodology, Supervision, Validation, Writing – review & editing. **Antonio Farina:** Resources, Validation, Writing – review & editing. **Maria Bastianelli:** Resources, Data curation. **Annalisa Cassardo:** Resources, Data curation. **Cristina Mei:** Resources, Data curation. **Antonio Lotti:** Resources, Methodology, Supervision, Validation, Writing – review & editing. **Luca Massacesi:** Supervision, Validation, Writing – review & editing. **Valentina Damato:** Conceptualization, Project administration, Resources, Methodology, Supervision, Validation, Writing – review & editing. **Antonello Grippo:** Conceptualization, Project administration, Resources, Methodology, Supervision, Validation, Writing – review & editing.

## Funding

VD is supported by #NEXTGENERATIONEU (NGEU) and funded by the Ministry of University and Research (MUR), National Recovery and Resilience Plan (NRRP), project MNESYS (PE0000006) - A Multiscale integrated approach to the study of the nervous system in health and disease (DR. 1553 11.10.2022), by a grant “Giovani Ricercatori – Ricerca Finalizzata 2021” code GR-2021-12375527 (“NEUROCHECKMATE”) from the Italian Ministry of Health and by a grant from the EJP-RARE DISEASE 2023 “OptiMyG”.

## Declaration of competing interest

VD has received public speaking honoraria and compensation for advisory boards and/or consulting fees from UCB, Alexion, Dianthus, Roche. She declares no competing interest regarding this manuscript. GS has received public speaking/consulting honoraria from Alexion, AMGEN and Argenx. He declares no competing interests related to this work.

## Data Availability

The data supporting the findings of this study are available within the article. The raw data are available from the corresponding author upon reasonable request and will be shared after appropriate anonymization in accordance with ethical and privacy regulations.

## Appendix A Supplementary data

Supplementary material: Supplementary Fig. 1. Local reference limits and cohort-derived diagnostic threshold for SFEMG parameters. (A) Mean MCD in healthy controls followed a normal distribution, and the upper local reference limit was defined as mean + 2 SD, corresponding to 26 μs. (B) Individual jitter values in healthy controls were not normally distributed; therefore, the 95th percentile of control values was adopted as the local upper reference limit, yielding 32 μs. (C) The proportion of endplates with jitter >32 μs was evaluated in the symptomatic cohort as a diagnostic classifier. The 27% threshold was derived from the clinical cohort to improve diagnostic specificity and should not be interpreted as a normative reference limit. Abbreviations: MG: Myasthenia gravis; MCD: Mean consecutive difference; SFEMG: Single-fiber electromyography. Supplementary Fig. 2 Diagnostic accuracy of SFEMG in identifying MG, assessed by ROC curve analysis. (A) ROC curve for mean MCD, with an AUC of 0.863 (95% CI: 0.793–0.934). (B) ROC curve for the percentage of endplates with abnormal jitter, with an AUC of 0.836 (95% CI: 0.762–0.911). Abbreviations: MG: Myasthenia gravis; MCD: Mean consecutive difference; SFEMG: Single-fiber electromyography; AUC: Area under the curve
