# Supplementary material for: Stimulated single-fiber electromyography in the diagnostic workup of diplopia and ptosis
Source: Clin Neurophysiol Pract. 2026 Sep 19;11:796–803. doi: 10.1016/j.cnp.2026.09.004 (PMC13633939; doi:10.1016/j.cnp.2026.09.004)
Supplement: Supplementary file 1 [file mmc1.pdf]

Supplementary Figure 1

A. Mean MCD

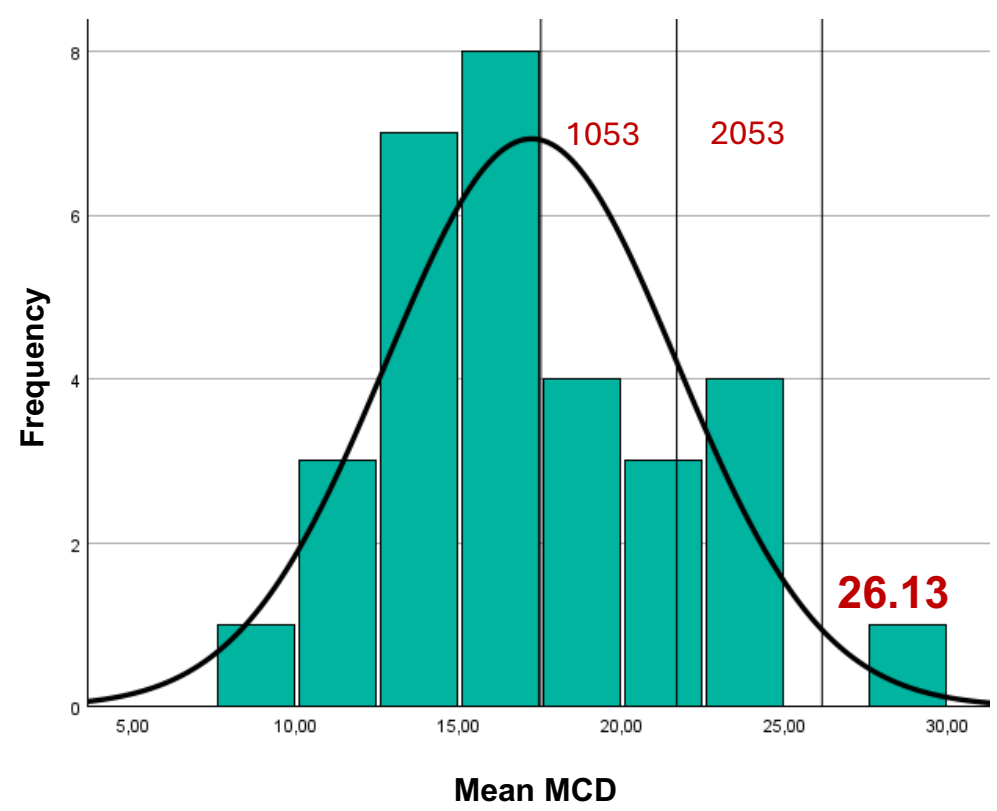

B. Individual jitter

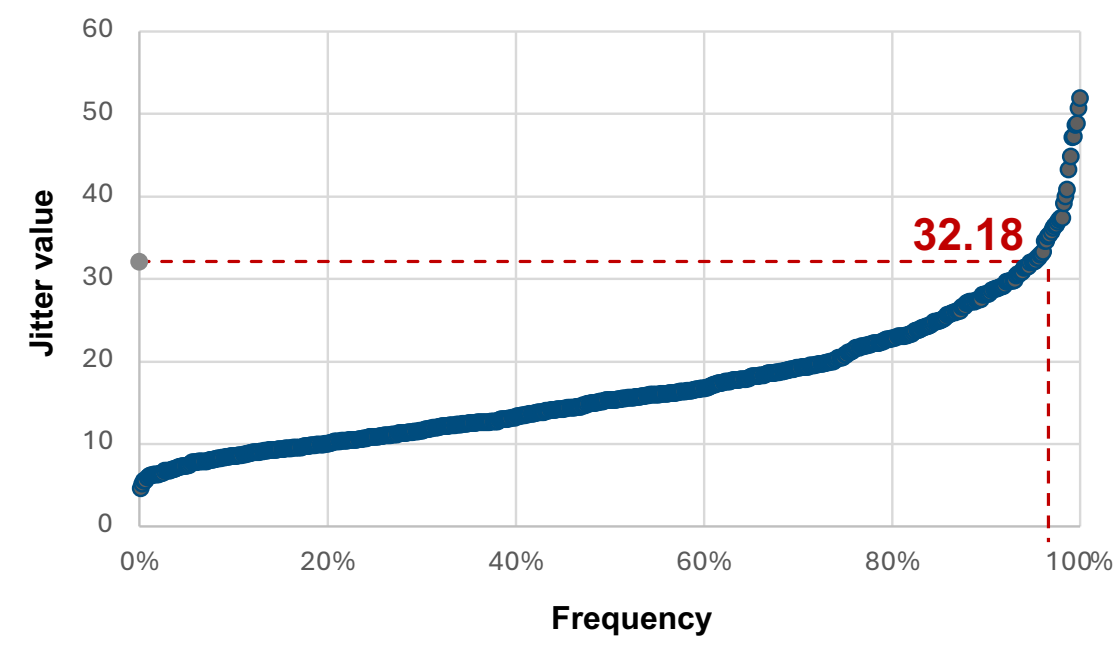

C. Percentage of endplates with abnormal jitter

C4

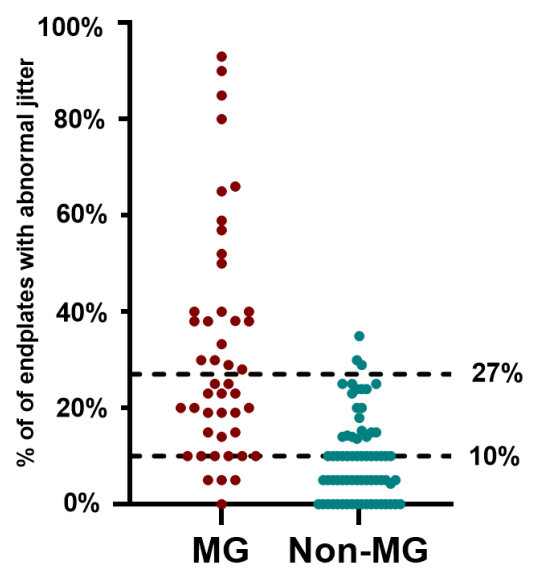

C44

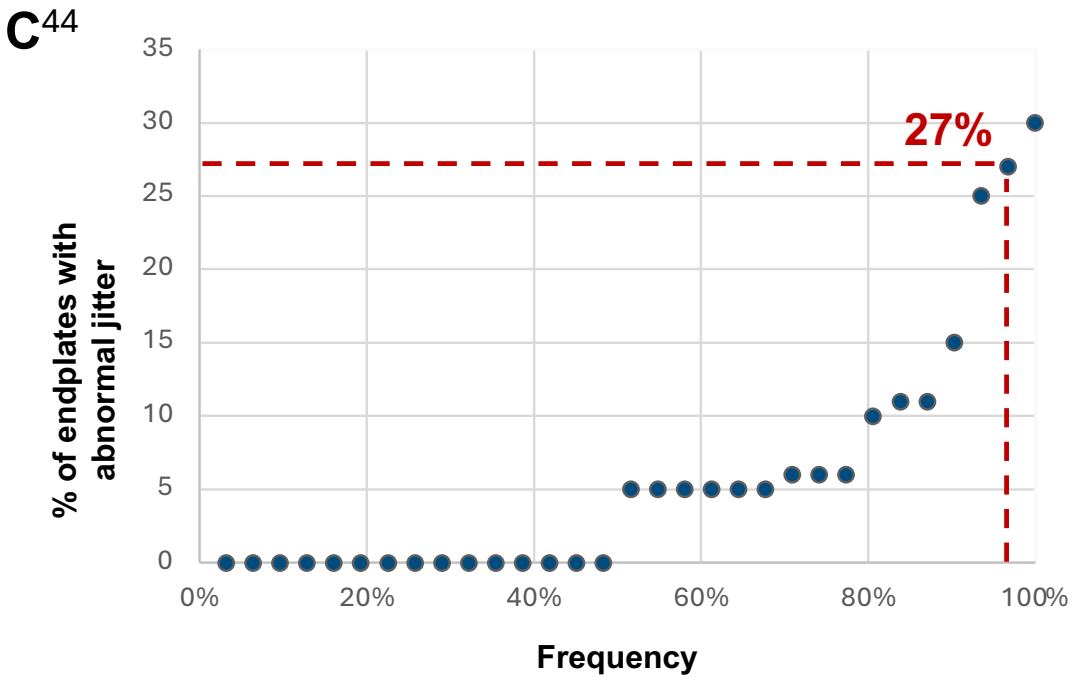

Supplementary Figure 2

A. Mean MCD

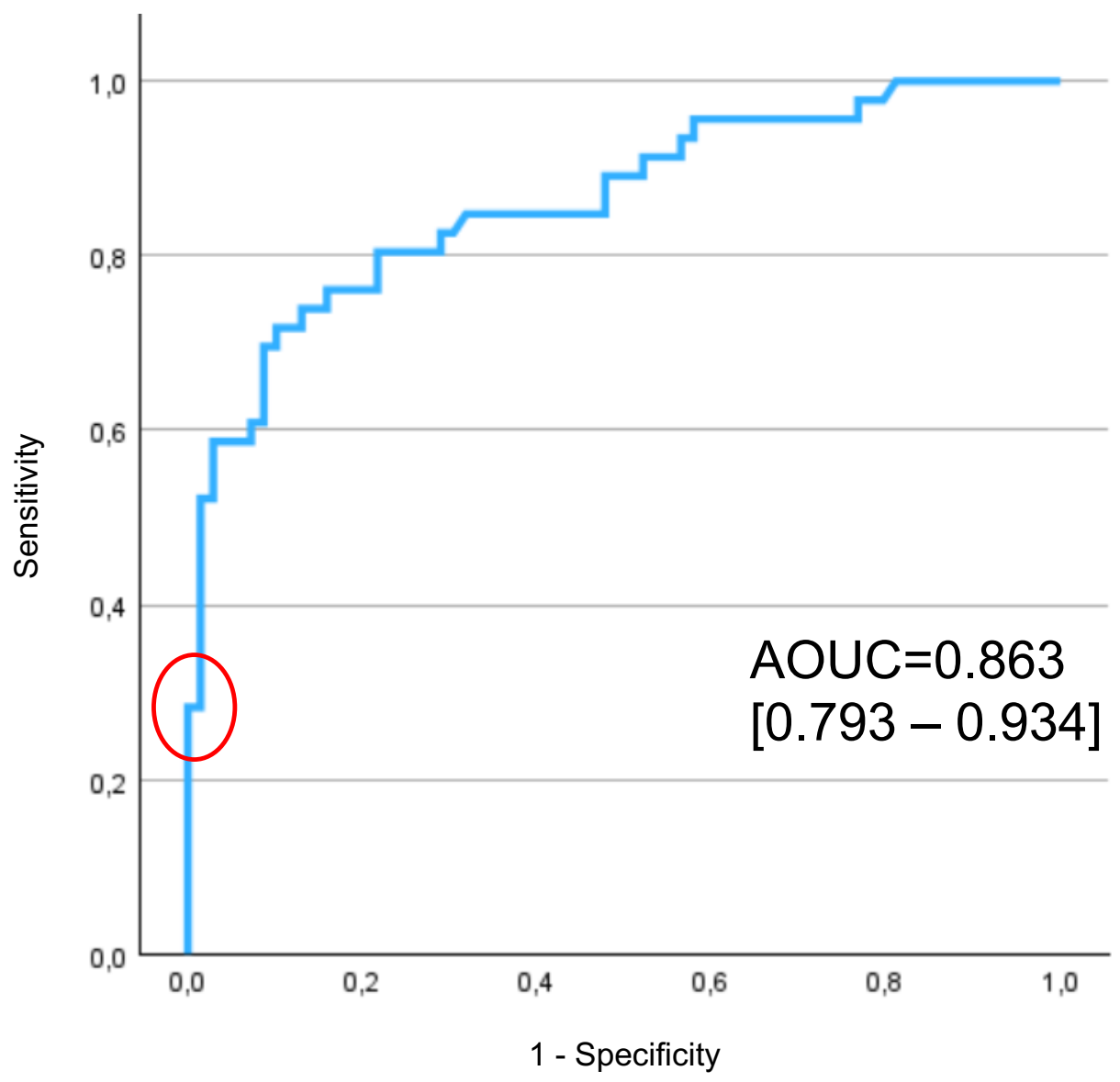

B. Percentage of endplates with abnormal jitter

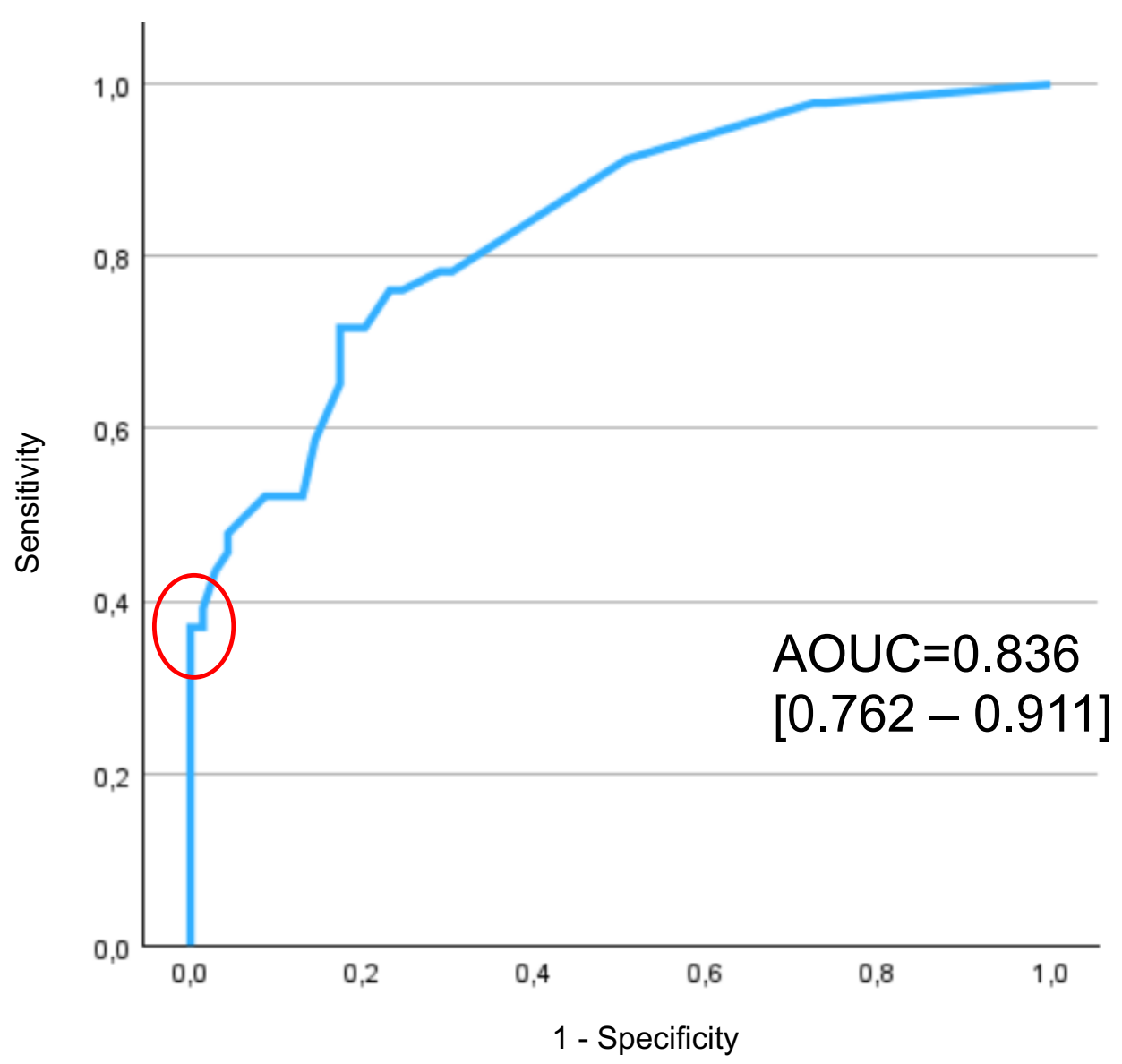

Supplementary Table 1. Diagnostic performance of SFEMG using the threshold values proposed by Sanders et al. compared with the thresholds calculated in the present study. Sensitivity and specificity are reported with 95% confidence intervals for both approaches.

	**Sensitivity**	**Specificity**
**Normative values calculated at AOU Careggi**	**60.9%**<br>(95% CI: 0.45–0.75)	**92.8%**<br>(95% CI: 0.84–0.98)
**Normative values calculated by Sanders and Stalberg** [REF]	**95.65%**<br>(95% CI: 85.16%–99.47%)	**53.62%**<br>(95% CI: 41.20%–65.72%).
